# Telehealth and Transformation of Nursing Care in Saudi Arabia: A Systematic Review

**DOI:** 10.1155/2022/8426095

**Published:** 2022-09-24

**Authors:** Ibrahim Al Baalharith, Mona Al Sherim, Sarah Hamad G. Almutairi, Azizah Saleh Alhaggas Albaqami

**Affiliations:** ^1^Nursing Adminstration, General Health Affairs in Najran – Ministry of Health, Saudi Arabia; ^2^Department of Surgical and Medical Nursing, College of Nursing, King Khalid University, Abha, Saudi Arabia; ^3^Department of Psychiatric & Mental Health & Community Health Nursing, College of Nursing, Qassim University, Qassim, Saudi Arabia; ^4^Department of Nursing Education, College of Nursing, Qassim University, Qassim, Saudi Arabia

## Abstract

**Introduction:**

Technological advancements have transformed nursing care, quality, and education across the globe. In the Kingdom of Saudi Arabia (KSA), the inventions and adoption of mobile technologies such as an e-health application (app) called SEHA continue to revolutionize the healthcare system in the country.

**Purpose:**

The present systematic review is aimed at examining the technological impact on nursing in Saudi Arabia. The study provides a comprehensive analysis of telehealth and its role in nursing quality, nursing practice, and education.

**Methods:**

The present study adopted a literature review methodology by deriving data from journal articles from different databases, for example, Web Science, Google Scholar, CINAHL, MEDLINE, and PubMed databases. Inclusive years for the search ranged from 2016 to 2022. A total of eight articles were found dovetailing to meet the research objectives and answer research questions.

**Result:**

After appraising and analyzing the research, the present review found that (Abolfotouh et al., 2019) telehealth in nursing is loosely researched; (Ahmed et al., 2021) telehealth impacts nursing practice and quality by fostering nurse-patient communication promoting positive outcomes, seamless nursing care, and positive experiences; and (Albahri et al., 2021) telehealth and telemedicine is a central tenet of contemporary nursing education and practice.

**Conclusion:**

From these findings, this analysis informed three key recommendations: the need to integrate telehealth into the nursing curriculum, telehealth training, and reskilling among healthcare workers (HCWs) in KSA and further primary studies focusing predominantly on telenursing. Overall, telehealth remains a fundamental transformation of nursing practice that forms a central ideology in the contemporary nursing process.

## 1. Introduction

Technological advances have transformed the medical landscape dwindling meticulous charting and manual filing. Telemedicine architecture includes communication through networks, artificial intelligence techniques, wearable sensors, smartphones, cloud computing, and hardware tools [1]. The integration of information systems and automated services has revolutionized the delivery of care. Primarily, healthcare professionals can provide remote clinical care, education, and health administration using fast and effective electronic information and telecommunication technologies [2]. In nursing, telehealth helps conduct nursing processes and deliver nursing care to remote clients across the globe. Telemedicine makes it possible for physicians to exchange various and durable patient information that can be used to track the health of patients living in remote areas [3]. Additionally, it has made it possible for the triage process in medical emergencies to be automated thus reducing the time needed to assess and classify patients into different groups depending on the severity of their condition [3]. With this, doctors are able to prioritize their rescue operations by taking care of the most critical patients first. The technology has transformed nursing by facilitating the accessibility of patient data, decreasing human error, and reducing burnout associated with nursing shortages. Telehealth helps improve patient satisfaction as it reduces delays in appointments, referral time needed to travel, transport costs, and time needed to be off work [4]. During the recent incidence of COVID-19, telehealth enabled patients and doctors to communicate through smartphones and computers with web cameras regardless of time and day [5]. Also, it enabled physicians to provide care to patients without the risk of exposure. With the advent of COVID-19, telehealth has been proven instrumental in promoting social distancing and advancing nursing technology. There is extensive literature evidence detailing the success of telehealth in specialty care, an aspect that has fostered the use of technology to transmit health data and provide care in mainstream healthcare service delivery [2, 6–8]. The major goal was to compile all telehealth researches in order to reveal the region's current telehealth research and innovation condition. The inquiry focused on federal benefits from telehealth, electronic health record deployment and satisfaction, digital technology in medical training, innovative systems, information security, and personal health records. According to the study, Saudi Arabia has a strong medical information system culture that encompasses all areas of telehealth. Future research should focus on the expense of eHealth projects, as well as religion and gender-related concerns in telehealth.

## 2. Research Gap/Problem Statement

Although a cohort of literature has examined cost-effectiveness, patient acceptance, and the role of technology in healthcare, there is scant evidence on the impact of telehealth on quality nursing care, practice, and education. This aspect informs the goal of the present systemic review, which seeks to provide a comprehensive, multifaceted analysis of telehealth in the context of nursing practice. Specifically, the present review examines the impact of telehealth and its role in transforming nursing care in Saudi Arabia. The study is divided into several primary sections, including background, methodology, results, discussion, limitations, and conclusions. It also sheds light on the most reliable recommendations that could help advance the eminence of care in Saudi Arabia using telehealth perspectives.

## 3. Background and Context

Telehealth encompasses all facets of remote healthcare services that include the provision and management of healthcare using computer-based technologies. The dynamic advancement in medical technology over the last decade has transformed the delivery and management of healthcare globally. Telehealth was coined in the 1970s to expound on the use of technology to improve patient outcomes through increased access to information and care [9]. In this vein, the Centre for Disease Control (CDC) defines telehealth as the “use of electronic information and telecommunication technology to get the health care you need while practicing social distancing” (2021). Conversely, Bashir & Bastola [10] define telehealth as “It is the integrated use of electronic information and telecommunications technology to support remote clinical health care, patient and professional health-related education, public health, and health administration” (35). The diverse nature of definitions posits that telehealth is a dynamically evolving medical and nursing science due to its ability to respond to changing technological advancements and adapt to changing needs and contexts.

The use of information and communication technology (ICT) in healthcare began in the 90s and has consistently evolved over the years. For instance, in 1920, radio was sued to give medical advice to clinicians on ships, while in the 1940s, NASA used closed-circuit television for telemedicine with the evolution of transmission of radiography [8]. Since the 1970s, the use of technological approaches to transmit medical advice or conduct a diagnosis has become a common occurrence in healthcare. For instance, in the 1980s, the advancement in telephones also saw its adaptation in delivering medical information [8]. While affirming these arguments, Lusting (2012) notes a high correlation between in-person and telehealth-based diagnoses. Today, telehealth is expanding in many countries, mainly with the desire to reduce healthcare costs. Nonetheless, the current epidemiological factors, such as increasing aging populations and the prevalence of chronic conditions, point to the crucial role of telehealth. Barbosa & Silva's [8] studies recognize the aggravation of infectious diseases and the emergence of global pandemics such as COVID19 as key causes of increased adoption of telehealth. In addition to natural or artificial disasters, these scenarios could hinder access to urgent healthcare services, amplifying the risk of a disease or injury to one's health. However, with telehealth, healthcare professionals can deliver safe, effective, and patient-centered care within seconds.

The tactics of accessing care and communicating with healthcare providers are constantly changing. Digital advancements in healthcare have expanded the alternatives for patients who want to actively participate in the treatment, management, or monitoring of their health. According to Downes et al. [11], innovations in telehealth have enhanced the concept of patient-centered care—a component that has allowed nurse-client collaboration in providing high-quality healthcare. Patients and their families who participate in collaborative care via ICT empower healthcare providers to make informed decisions that reflect patient preferences and result in satisfaction. As a result, in the ever-changing and complicated healthcare systems, healthcare workers (HCWs) are continually considering telemedicine as a viable care delivery framework [12]. Besides, the technological growth and change in consumer behavior have led to the increased presence of technologically savvy patients. Such service users need efficient healthcare at the ‘tap of a finger, motivating developments in ICT for healthcare literacy and service delivery [12]. Thus, technology, in various forms, has been integrated into healthcare to meet current emerging needs.

Nursing forms a crucial facet of healthcare globally. According to the Nursing and Midwifery Council (NMC) [13], nurses recognize and respond to patients' needs using evidence-based technologies and pharmacological and therapeutic interventions. The profession remains identifiably the most versatile field in healthcare. Since Florence Nightingale developed the modern nursing theory to expound on the interplay between nursing, patient environment, health, and basic needs of an individual [14], nursing has strived to configure the patient environment to foster positive outcomes. The use of ICT is an evolving role of nursing in modern healthcare systems that have embraced home settings. According to Bashir & Bastola [10], telehealth empowers nurses with the ability to monitor, educate, collect data, follow up, and offer multidisciplinary care. These aspects are necessary for long-term wellness, self-management (autonomy), and innovative social support. The American Nurses Association (ANA) further posits that telehealth underlies the future of nursing by closing the rural-urban divide, providing health literacy remotely, and expanding access to much-needed healthcare (2021). Nonetheless, ANA recognizes telehealth as a fundamental facet of modern healthcare by facilitating remote diagnosis, supporting self-care among patients, offering continuous yet less costly monitoring, and leveraging the knowledge of nurses across a wide population of patients.

In Saudi Arabia, healthcare technology was first adopted in the 1980s with the introduction of electronic health systems [6]. The Ministry of Health sought to embrace technological advancement to enhance healthcare systems and patient outcomes. In 2002, the country embraced electronic medical records, an electronic platform for managing and organizing patient information [6]. Such advances have been steered through the national transformation programs started by the government to revolutionize healthcare and other sectors. A survey conducted later in 2011 established that at least 16% of hospitals used EHR systems effectively in the country's eastern region [7]. The increasing demand for healthcare services has necessitated the adoption of information and communication technology to cater to the needs. According to Alshammari [2], the average doctor-to-patient statistics in Saudi Arabia is at least 3 in every 1000 people. Reports indicate that the country might need to employ more health professionals as the population is expected to hit 45 million by 2050 [2]. These aspects inform the numerous initiatives the ministry of health took to increase ICT for the provision of healthcare services across Saudi Arabia. For instance, the Saudi Telemedicine Network (STN) was successfully launched to foster the adoption of telemedicine in KSA. Moreover, the adoption of the SEHA-mobile application has cemented efforts to enhance remote healthcare delivery in KSA.

In Saudi Arabia, the concept of telenursing is still under investigation. Most of the medical literature to date has discussed the advantages and disadvantages of various forms of telemedicine and telehealth. Research on the adoption and acceptance of telehealth in medicine and the practice of telemedicine among nurses has been conducted. Using telenursing as a concept in the context of quality nursing care, the transformation of nursing, and the impact on education or practice are all aims and objectives of this systematic study. As a result, such research is scarce when it is focused solely on Saudi Arabia. Existing research shows consistent telenursing themes such as quality of care and nursing practice. To find out how telehealth deployment can improve nursing practice, education, and care quality, this systematic review draws on these kinds of research studies. Telehealth poses extensive benefits in healthcare, including enhanced accuracy in documentation, quality care for the needy, and improved workflow, helps avoid errors, reduces the cost of care, and supports interprofessionalism in nursing. These key facets make telehealth a fundamental technology in contemporary healthcare settings.

## 4. Significance of the Review

The eHealth strategy benefited healthcare providers, healthcare managers, policymakers, health professionals, and patients. According to Chowdhury et al., [15], eHealth has the potential to improve the quality of healthcare systems (by reducing errors, enhancing diagnosis and treatment, and expediting healthcare decisions) and for healthcare professionals (by increasing information exchange among healthcare workers, reducing costs and time, and enhancing the safety culture among primary care providers). To reach the Saudi National Transformation Program (NTP) 2030, the MoH must achieve 15 objectives. The third objective of the Ministry of Health's plan is to boost the healthcare sector's efficiency and effectiveness through information technology and digital transformation [15]. The Ministry is aiming for at least 70% of the citizens to have unified digital records by 2030. Saudi Arabia seeks to grow its public sectors, such as education and healthcare, in order to improve the country's residents and citizens' quality of life. In order to achieve these objectives, Saudi hopes to transform most services to be accessed through electronic means, focus on training and qualifications, and open ambitious investment platforms. According to Saudi Vision 2030 (2016), these efforts are among Saudi's strategic planning to facilitate access to health services which can be done through eHealth services.

Saudi Arabia's Ministry of Health (MOH) implemented numerous forms of eHealth as a substitute for face-to-face consultations in clinical settings. Over 19 mobile apps have been launched to connect 96 percent of the population with a smartphone to web-based health services. Moreover, Saudi Arabia has deemed digital health a top priority for developing health services in the Kingdom. A group of projects has been launched in this framework, including the “Mawid” application, which provides a central appointment reservation service [16]. About 14 million people have signed up for this application, and more than 60 million appointments have been booked through it in 2020 (MOH, 2020). The evaluation will be essential in highlighting the issues that Saudi Arabia's nursing profession is now facing. Furthermore, the research will demonstrate how technology may enhance the nursing profession and assist in resolving current issues. Nurse shortages, burnout, and cultural differences are among the current problems [10]. The workload will be decreased due to the use of current technology, which can perform various tasks. There will be less risk of hospital-acquired infections due to nurse burnout if technology fills in some of the duties currently handled by nurses [17]. Background, methods, results, discussion, and limitations are all included in the study's main sections. According to this study, telehealth in Saudi Arabia can also improve the quality of care.

## 5. Review Objective and the Questions

The overarching goal of this systematic review is to critically analyze, consolidate, and present the available information on telehealth implementation and acceptability in Saudi Arabia from diverse stakeholders' viewpoints. Three research questions are proposed as part of the review:

1. What are the perspectives of Saudi Arabia's health experts, health IT specialists, and healthcare managers on the state of telehealth?
2. From the views of medical practitioners, healthcare IT experts, and health professionals, what variables influence telehealth implementation and acceptance in Saudi Arabia?
3. From the perspectives of health practitioners, health IT specialists, and healthcare managers, what barriers and facilitators are used for the implementation of telehealth in Saudi Arabia?

## 6. Methodology

The applications of technology-related issues varied in KSA; various technologies were used to introduce telehealth. The COVID-19 pandemic put pressure on telehealth capacity, resulting in limits such as limited insurance contracts, high training expenses, poor reimbursement, insufficient equipment, and software constraints; telehealth support has reduced the number of required adjustments and limits, allowing telehealth to expand more quickly. The review is written using the Reporting Items for systematic Elements for Meta-Analysis Protocols (PRISMA-P) and Systematic Review checklist of items before commencing this systematic review. PRISMA-P is a “guidance to assist writers in the preparation of protocols for planned meta-analyses and systematic reviews by providing a minimal set of things to include in the protocol” [18]. Many stakeholders, particularly reviewers, authors, and potential readers, will benefit from improvements in the quality, accuracy, and integrity of protocol material, as well as increased awareness of the minimal content for protocol reporting.

### 6.1. Design

This systematic review adopts a systematic review methodology that relies on an in-depth analysis of primary studies supporting the research topic. The methodology adopted is based on one fundamental technique, the Preferred Reporting Item to Systematic Review and Meta-Analysis (PRISMA). According to [18] PRISMA provides a framework for reporting systematic reviews by analyzing interventions, prevalence, diagnosis, or aetiologias. Moreover, the framework ensures that the systematic review is well organized and explicit to promote integrity, transparency, and focused review. Liberati et al., [18] maintain that PRISMA provides strategies for the exclusion and inclusion of studies, which helps in the identification of quality research papers.

### 6.2. Search Strategy

While employing the PRISMA approach, an advanced full search was conducted in different databases, for example, Web Science, Google Scholar, CINAHL, MEDLINE, and PubMed databases, and focused on two major electronic databases on 25th January 2022 using the English language. The advanced search relied on MEDLINE (PubMed) and Cumulative Index to Nursing and Allied Health Literature (CINAHL). The databases offer extensive alternatives to the literature pool with enough evidence on the research topic. Besides, the databases offer reliable, authentic, and peer-reviewed research papers. For instance, CINHAL offers over 4000 nursing and nursing science and allied indexed journal articles. It is a renowned health research repository with resources from around the world, including Saudi Arabia. On the other hand, MEDLINE is considered the largest medical science repository, with thousands of research papers, reports, and reviews. It provides an extensive list of free-access, peer-reviewed, and easily accessible research articles. From this end, the two databases proved instrumental in sourcing data about telehealth and its impact on nursing. The advanced search adopted several keywords to generate specific results. The keywords were generated from the research question and topic and joined using Boolean operators “OR” and “AND” to facilitate the return of sought results in either MEDLINE or CINHAL. The keywords and Boolean operators included “Telehealth” AND “Nursing in Saudi Arabia,” “Telenursing” AND “Saudi Arabia,” “Telehealth” OR “Telemedicine” AND “Saudi Arabia,” “Telehealth” OR “Telenursing” AND “Nursing Quality,” “Telehealth” AND “Nursing Practice,” and “Telehealth” AND “Nursing Education.” The use of keywords in and search tab in each database eased navigation in the databases and identification of desired research articles. After all, keywords form crucial facets in guiding focused, systematic review molded on research question, objectives, and methodology.

### 6.3. Inclusion–Exclusion Criteria

The existing literature presents evidence spanning a wide dimension of telehealth, telemedicine, and specifically telenursing. Such comprehensive data offers hundreds of sources that deviate from the intended results necessitating an exclusion and inclusion criterion. The criterion was provided for the inclusion of studies that were most relevant to the research question. The articles included met the following criteria: [19] primary research papers, [20] studies written in English, [1] published between 2016 and date, [21] peer-reviewed, and [22] articles that discussed telehealth with a focus on nursing in Saudi Arabia. Articles that were excluded failed to meet this criterion and included papers older than five years, systematic reviews/meta-analyses, reports/manuals/editorials, and those using a different language.

### 6.4. Data Extraction

The articles were selected based on different attributes, as shown in Table 1. These attributes were included because they were important in achieving the systemic review's key points. The purpose of the article title was to help in future reference. Challenges were used to highlight the various problems and concerns encountered in the past research. The motivations were used to highlight past studies' benefits and relevance. The recommendations are used to show the perspectives of past authors in future studies and discussions. The publication highlights how telehealth has been used in different care settings. These were the main points considered when writing down the taxonomy.

**Figure 1:**
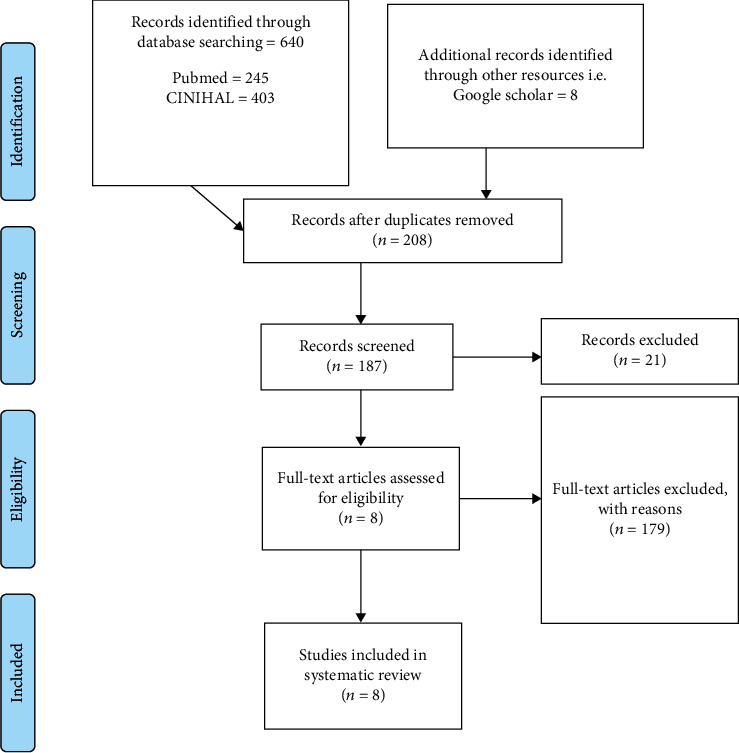
PRISMA flow chart showing the search process.

After examining and identifying the subset of primary literature that met the inclusion criterion, data were extracted from the articles into a table. This included basic information about the articles, including author, date, purpose, methodology, findings, and main conclusions. Moreover, the extraction focused on themes or subthemes related to telehealth, telehealth technology, and the function telehealth played in nursing care. To develop the tables, a list of articles and information of interest was designed (see Table 2). The list was tested, refined, and finalized in tandem with a research question, problem statement, and PRISMA model. A list of included studies and their basic information, including sample sizes, was also generated (see Figure 1). Such a list helped ensure the elimination of duplicated studies, double-counting, and ensure reporting of primary studies answering the research question.

### 6.5. Data Analysis

The present study will systematically examine and analyze articles for quality, reliability, and relevance in telehealth. Evidence-based information provides the best evidence to deliver safe, quality, and effective healthcare that meets clients' preferences and improves outcomes. Nursing and Midwifery Council (NMC) [13] argues that evidence-based nursing practice embraces the best available nursing evidence that considers professional experience, available technologies, and preferences of service users. While most studies are geared towards such an outcome, literature evidence varies widely in terms of quality, grading, and reliability. This study adopted the Cardwell appraisal framework to determine the efficacy of the included data. Primarily, quality assessment was conducted using the Cardwell framework, which examines qualitative and quantitative studies. Cardwell et al., developed the framework in 2011 to systematically review the quality of studies using checklist items. The framework identifies and reviews common features in the two research methodologies to determine the most reliable research. This aspect informed the need to embrace eight key criteria from the Cardwell framework. These included the title and abstract, methodology, sample size, the purpose of study and rationale, findings, and ethical approvals, as shown in Table 3. Studies that met all the eight criteria were considered having quality, while those that met at least six of the criterions were considered having moderate quality, while any study meeting less than six was considered poor quality and excluded. The research findings are represented in terms of themes to ensure they capture all relevant data.

### 6.6. Results/Findings

The initial generic search in the two considered electronic databases (MEDLINE and CINAHL) resulted in 648 hits, 245 from PubMed and 403 from CINAHL. As a result, additional filters were included to single out eight studies exclusively. First, duplicated studies were removed, reducing the number to 208 articles (n-440). Secondly, a date or year filter was used, plummeting the number to 92 by limiting the search to studies conducted within the last five years (n-116). Thirdly, studies using a language other than English presented analysis problems and were excluded reducing the number to 40 (n-52). Articles were then examined for the title and abstract relevance, which reduced the number of articles by 21 (N-21 = 19). Conversely, 11 articles were eliminated due to limited access to the full article, and eight studies were found dovetailing to the research question and purpose. Thus, they were included in the research and considered for full-body analysis. Figure 1 below represents the PRISMA flow chart, which expounds on the inclusion and exclusion criteria adopted to arrive at the eight studies. Nonetheless, Table 4 represents the evidence from the eight studies.

### 6.7. Critical Evidence Appraisal

The dynamic advancement in technology is transforming healthcare and the practice of nursing. It is recommended that evidence-based nursing practices adhere to explicit and judicious use of the most current evidence. According to the Nursing and Midwifery Council (NMC) [13], the standards of competence are applied across all nursing practices as guided by interpersonal skills, technology, and professional values. The competencies revolve around extensive yet effective professional use of available evidence and technologies that inform the practice. While thousands of literature materials have been published to inform nursing practice and transformation in nursing care, the evidence tends to vary in terms of reliability, quality, and grading [26]. As a result, the present study relies on a framework developed by Cardwell, Henshaw, and Taylor in 2005 and updated in 2011. The framework provides a judicious approach to crucially analyzing qualitative and quantitative research papers by relying on similar aspects in these studies relevant to quality research critique. In tandem with Caldwell (2011), this study will use twelve essential checklists including to critique and determine the quality of incorporated studies.

The selected eight studies [2, 19–25] used the single sentence, explicit, and informative titles that provided information about the purpose of study, location, and target population. Titles ought to comprehensively reflect the content of the study in a precise yet explicit manner. Primarily, a title should guide the reader by providing enough information about the study to determine its purpose. Conversely, the eight papers used adopted a reliable abstract comprising the research aim, background, methodology, results or findings, and conclusions.

The rationale tries to expound on the core reasons being a given study. Rationale connects the research question and problem to the findings ensuring the purpose is met. Included studies met this criterion by presenting a problem statement to exhibit the need for the research. Alshammari [2] recognized the dissimilarities in attitude, experiences, and perception between urban and rural service users towards telehealth and conducted a study to examine the extent of understanding of telehealth among the general KSA population. In the same vein, Ahmed et al., [20] identified limited acceptance, understanding, and experiences among young nurses and doctors in Kind Abdulaziz University Hospital (KAUH), Jeddah. These aspects informed the need to research to either affirm these allegations or provide evidence proving otherwise. Similar aspects led Abolfotouh et al., [19] to study the use of smartphones to facilitate remote healthcare among healthcare workers IN KSA. Alfaleh et al., [21] noted the government efforts toward medical technology such as mobile application SEHA in over 900 medical centers. The researcher conducted a survey to examine the impact of the technology, especially given the high satisfaction rate among service users. Conversely, the emergence of COVID-19 and accompanying medical technologies to facilitate remote healthcare while promoting social distancing evoked ALOmari, and Jenkins, J. [23] need to examine the attitude and experiences of patients using SEHA. Alkamel et al., [22] examined stakeholders' opinions and the expectations of healthcare workers working with ST-elevated myocardial infarction (STEMI) patients. Most healthcare workers are not usually willing to embrace such technology. This can be due to limited knowledge or sheer ignorance. Thapa et al., [24] sought to determine the willingness to use telehealth among HCWS in KSA. While other research papers focused on telehealth and its impact on healthcare workers and service users, Al-Marashi and Al-Zghool [25] took a different approach by examining issues that affect job performance among nurses. The study helps build on the need for telehealth, as affirmed by the other seven studies included in this research. Based on this evidence, none of the studies focused exclusively on how telehealth transforms nursing practice, an aspect that builds the rationale for the present systematic analysis.

To this end, the framework questions the identification of conflict of interests, ethical approval, and informed consent. Heale and Shorten (2017) affirm the criterion by noting that ethical concepts such as autonomy, beneficence, justice, and nonmaleficence must be recognized in research. The included studies fulfilled the concept of autonomy and justice by seeking informed consent from participants. Since autonomy builds on the ability to make decisions independently without influence, these studies met the ethical criterion. Conversely, the research purpose suggests that it sought to benefit healthcare workers, stakeholders, and general populations—an aspect considered as beneficence. Lastly, nonmaleficence builds on autonomy and justice, ensuring participants are protected. Research methodology is another critical facet integrated into Cardwell et al.'s framework of research critique. The methodology provides an approach to identifying, analyzing, and presenting research data in tandem with identified research questions, hypotheses, and problem statements. The present analysis relied on qualitative and quantitative research methods, which provide primary data crucial in systematic reviews. Quantitative research focuses on numerical data, while the qualitative approach relies on audio, visual, text, or experiences as key sources of data. The appraising methodology remains instrumental, given that comprehension of research is a prerequisite in determining the strengths and weaknesses of a study. Research by Ahmed et al., [20], ALOmari, and Jenkins, J. [23], and Alkamel et al., [22] used qualitative data. The qualitative research is crucial in healthcare given its ability to answer the question “WHY” a given phenomenon exists and “HOW” it impacts healthcare systems or service users. On the other hand, Thapa et al., [24] and Alfaleh et al., [21] adopted a quantitative methodology, which helps collect and integrate data collected through scientific inquiry into nursing science. Solvik and Struksnes [27] argue that quantitative analysis helps measure specific variables in healthcare and provides precise data. Given that primary data is collected through an experimental approach, observations, experiences, measurement of variables, and text analysis, these studies satisfy Cardwell's framework of research critique.

Sample sizes are the last facet of the adopted checklist. Primarily, sample sizes help to determine whether the findings are reliable and can be generalized to different settings, demographics, or populations [28]. Large sample sizes offer proximate mean appropriations with relatively small margin errors in addition to the ease of generalizing the research results. The research by Alshammari [2] included 781 participants representing the general population in KSA; Ahmed et al., [20] relied on 335 HCWs; Alfaleh et al., [21] included 319 participants; Abolfotouh et al., [19] relied on a sample of 351, and Al-Marashi and Al-Zghool [25] included 344 nurses. These studies are considered strong and reliable. Conversely, samples with less than 25 participants may be considered small, especially when it comes to the limited generalizability of results. Such raises concerns about two included studies, ALOmari, and Jenkins, J. [23] and Alkamel et al., [22], which used a sample of 17 and 22 participants consequently. Despite this, Bolarinwa's [28] research alleviates the concerns by asserting that small sample sizes are instrumental in situations where sample sizes do not affect the outcome. Besides, small sample sizes tend to minimize the costs of conducting research.

## 7. Findings

### 7.1. Impact of Telehealth on Nursing Practice

A cohort of literature has examined the relevance of telehealth in healthcare. The National Health Information Center (NHIC) [29] presented general guidelines for telehealth that majored in the transformation of HCWs and patient relationships. Primarily, the technology eases communication by facilitating seamless teleconsultations between the healthcare worker and service user. This research finds a high preference for telehealth consultations among service users and healthcare professionals. Alshammari [2] found a 70% preference among the KSA population for telemedicine consultations on healthcare issues, while ALOmari, and Jenkins, J. [23] found that 76% of the participants regularly used telemedicine services. This aspect specifies the transformation n service users, which directly indicates a shift in nursing practice. For example, Abolfotouh et al., [19] found that 42.3% of healthcare workers used smartphones in the healthcare practice. While examining the attitude of nurses and doctors using telehealth in their practice, Thapa et al., [24] established that over 75% of the participants were willing to use telehealth and 62% had experience using telemedicine at departmental levels. This systematic review finds extensive adoption of telehealth in nursing practice.

### 7.2. Impact of Telehealth in Education

The research identifies the adoption of telehealth in nursing education. Ahmed et al., [20] found that over 90% of nursing students use Internet-based technologies for health-related technology. Moreover, the authors identify that the students understood and preferred using mobile apps in their nursing practice. For instance, Thapa et al., [24] found that nursing students were enthusiastic about the use of telehealth for remote monitoring, accessing health information, and follow-up. In separate studies, Bashir & Bastola [10] noted that technology is a crucial component of nursing education. The authors found that nursing students exhibited a high preference for telehealth.

### 7.3. Telehealth and Quality Care

All the studies associated telehealth with improved healthcare practice. The technology helps develop the patient-centered nursing practice by facilitating nurse-patient interactions. Abolfotouh et al., [19] found that telehealth helped forge a close relationship between the patient and healthcare provider, which further helped people to develop trust and acceptance of the technology. Moreover, Alfaleh et al., [21] found that developing and applying the SEHA mobile app in KSA have helped reduce work overload in emergency departments. Primarily, over 24% of patients changed their minds about visiting the ED after consulting using the SEHA app. While building on these findings, ALOmari, and Jenkins, J. [23] found that telehealth helped reduce person-to-person contact during the COVID-19 pandemic, in addition to proving instrumental in emergency services. These aspects indicate the impact of telehealth in practice.

## 8. Discussion

The high nursing shortages and increased burnout are key aspects fostering the constant adoption of technology in nursing care. Moreover, nursing focuses on patient-centered care that necessitates the use of different strategies and technologies that facilitate nurse-patient communication. Nursing and Midwifery Council (NMC) [13] requires nurses to embrace a wide array of strategies to facilitate respectful, empathetic, and effective communication with patients that facilitates positive patient outcomes. The present systematic review finds telehealth as an instrumental technology in healthcare, especially during epidemics [23] or when handling specific conditions such as STEMI [22]. All the included studies consider telehealth as a crucial facet of healthcare. An earlier study by Aloraini [30] presented dissimilar evidence after finding the limited significance of telehealth in ICU patients. However, the research by Al-Marashi and Al-Zghool [25] considers burnout, workload, and limited innovations as factors hindering job performance among nurses. These firings support the need to integrate innovative technologies in nursing to alleviate such challenges.

The findings suggest that technology remains a central tenet of nursing practice based on its ability to promote patient-nurse contact. The study by Thapa et al., [24] finds a 70% acceptance rate among nurses. Moreover, Ahmed et al., [20] found that HCWs, including nurses, had a comprehensive understanding of telehealth, while Alkamel et al., [22] found a high preference for telehealth services among HCWs. Some of the findings align with previous and current research from various countries. Alkhashan et al., [31] conducted a study to determine the rate and satisfaction of HCWs and service users relying on telephone consultations. The authors found that people in KSA used SMS, audio, and video class to conduct consultations with doctors, pharmacists, nurses, and other professionals. Bashir & Bastola [10] affirm these findings by noting that telehealth effectively helps nurses manage chronic illness patients. Through telenursing, nurses focus on long-term wellness, health, and autonomy. It provides an opportunity for a nurse to constantly monitor, follow up, or work with patients either in the healthcare facility or at home.

With the advent of COVID-19, telehealth has proven instrumental in promoting social distancing and advancing nursing technology [23, 32]. In many hospitals, patients were referred for home care due to congestion in hospitals and the need for social distancing measures. In this vein, telehealth provided an opportunity for nurses and other healthcare workers to provide much-needed care from remote locations [32]. ALOmari, and Jenkins, J. [23] note that the SEHA application (telehealth) saved time and costs for patients during the pandemic while offering the service users an opportunity to access professional services. A study by Monaghesh & Hajizadeh [33] affirms these findings by noting that remote healthcare reduces the risk of transmitting infectious diseases and reduces the usage of resources among HCWs and service users. The platforms also provide wide access to healthcare during the pandemic offering an identifiable contribution to nursing practice.

The Kingdom of Saudi Arabia has experienced a boom in healthcare in the last few decades. The advancement in nursing and medicine practices provided by thousands of hospitals and community centers can reach people in remote areas through SEHA and other telehealth technologies. In line with such advancements, technology continues to revolutionize nursing education to meet contemporary demands. Ahmed et al., [20] and Thapa et al., [24] found a high acceptance of telemedicine among nursing students indicating the potential transformation of nursing practice. A cohort of literature indicates that telehealth can be used to provide education, enhancing health literacy among the public ([34, 35]: [25]). These aspects indicate improvement in nursing care and quality.

Findings depict that telehealth enhances the quality of nursing practice. Ahmed et al., [20] found that nurses and doctors were enthusiastic and preferred using telehealth to manage patients with chronic conditions. The technology enhances the ability to monitor and respond to patients' needs immediately without the need to visit healthcare facilities. However, the study by Aloraini [30] failed to establish any positive association between telehealth and reduced mortality in the ICU. However, more significant findings were identified by Bashir & Bastola [10], who noted that telehealth substantially improved nursing quality by facilitating communication, management of the service user, autonomy among patients, and ease in monitoring or following up on the patients. These assertions also reflect the findings obtained by Thapa et al., [24], Alkamel et al., [22], and Abolfotouh et al., [19], who noted the high preference for mobile applications or ICT in the management of conditions or conducting healthcare practices. The study by Al-Marashi and Al-Zghool [25] finds nursing to be a challenging profession characterized by burnout and high workload. These aspects can be mitigated by adopting telehealth to facilitate seamless care for patients in KSA.

## 9. Limitations of the Study

Although the present systematic analysis provides useful insight into the transformation of nursing practice by extensive technologies such as telehealth, there are limitations that could raise concerns. First, the present analysis relies on only eight studies, which limits the extent of findings and drawing conclusions. Secondly, there is scant literature examining the transformative nature of telehealth with a specific focus on nursing in KSA. Current findings thus rely on evidence focusing on general HCWs in KSA. Some of the inherent issues of the individual studies, such as sample sizes souls, also are considered when interpreting the results of this systematic review. Lastly, the review is exclusively based on research conducted in the KSA, which could limit generalizability in the international or regional context.

## 10. Recommendations

This systematic review finds evidence of the significance of telehealth in mitigating challenges in nursing, promoting quality care, and facilitating education. These lay the rationale for the following recommendations:

- The nursing education curriculum should integrate telehealth or telenursing studies to ensure all healthcare workers have relevant knowledge about the technology. Research by Alkamel et al., [22] and Ahmed et al.
- , [20] finds that several HCWs do not understand telehealth, although over 80% of the professionals had an idea about the technology. Given the Nursing and Midwifery Council (NMC) [13] requirements to promote quality nursing practices built on competence, integrating telehealth training into the curriculum could help impart the skills to student nurses
- Hospitals in KSA should enhance telehealth training and facilitate the adoption of the technology. Primarily, all countries, including KSA, face a significant shortage of nurses and other HCWs. Investing in telehealth could help reduce the effects of nurse shortage by allowing a nurse to easily access patient information, provide patient education, and promote autonomy through telehealth to more than one patient
- There is limited primary research on telehealth and the transformation of nursing practice. Thus, researchers should focus on this area to provide more precise evidence on the impact of telehealth on nursing practice

## 11. Conclusion

This study comprehensively analyzed several studies to highlight the significance of telehealth in nursing and healthcare in general. Based on the analysis findings, nurses in KSA face challenges associated with burnout and workload, which can be mitigated using telehealth. The ability to mitigate such challenges helps enhance the quality of care by fostering satisfaction among nurses. There is variable evidence to suggest that nurses in KSA understand telehealth, especially given the implementation of the SEHA app to facilitate remote healthcare in the country. Telehealth helps nurses forge relationships with service users by creating a communication medium. Such aspects help to foster patient-centered care. This aspect has helped augment healthcare quality through self-management and improved patient outcomes. However, more evidence with higher quality and stricter methodology is needed to clarify the long-term impact. This systemic review provides up-to-date proof of the impact of telemedicine in healthcare and offers objective information for institutions intending to use telehealth to improve patient care. This solution should be considered to address the issues presented in the article. A significant relationship exists between the requirement for telemedicine and its use in the current situation in healthcare. Telehealth should be properly integrated into healthcare to help physicians and policymakers improve medical care practice to the highest standard.

## Figures and Tables

**Table 1:** Data extraction and classification contents.

Item	Description
Title	Article title
Year	Article's publication year
Challenges	Different concerns and problems are highlighted in the article
Motivations	The publication's benefits and significance
Recommendations	Identification of future directions or advisements in the article
Nature of application	How telehealth was implemented and used

**Table 2:** Search terms used in the search strategy.

Keywords table	
Telehealth	Nurse education
Telemedicine	Nursing practice
Nurs∗	Saudi Arabia
Transformation of nursing	
Quality care	
Tele∗	

**Table 3:** Quality assessment and selection criteria.

Criteria for quality assessment	
Design	(i) Does the abstract provide sufficient information? (ii) Does the study clearly state its objective? (iii) Does it describe its method clearly? (iv) Are the research methods used effectively in attaining the research objectives? (v) Is the study setting and used sample justified and reproductive? (vi) Does the study fully define its evaluation metrics? (vii) Are the utilized evaluation metrics significant for the research?

Data acquisition	(i) Does the research describe its data collection method adequately?

Data analysis	(i) Does the study describe its data analysis adequately? (ii) Does it compare its results with that of other studies?

Conclusion	(i) Does the study clearly state its findings? (ii) Do the results support the findings? (iii) Does it highlight its limitations?

**Table 4:** Summary of study characteristics and extracted information.

#	Author	Date	Purpose/rationale	Methodology	Sample	Major findings
1	Alshammari [2]	2019	It aims to assess service user perception and preferences about telemedicine	(Mixed method) cross-sectional study	*N* = 781 people	70% acknowledge the benefits of telehealth
						52% have never used telemedicine such as Sea
						29% exhibited a lack of trust toward telemedicine/telehealth
2	Ahmed et al., [20]	2021	Investigate nurses' and doctors' perceptions, knowledge, and attitude toward telemedicine for follow-up monitoring of chronic conditions	Qualitative-survey	*N* = 335 professionals	Participants exhibited positive attitudes and perceptions toward telehealth
3	Alfaleh et al., [21]	2021	Role of telemedicine in reducing nonurgent visits to the emergency department	(Quantitative) cross-sectional study	*N* = 319 patients	Use of telemedicine reduced visits to the ED
4	ALOmari & Jenkins et al., [23]	2021	Examine the attitude of patients towards telehealth-Seha	Qualitative-grounded theory	*N* = 17 patients	Concerns about technical or misdiagnosis
						Telehealth is a crucial tool for working with patients in a pandemic
						Telehealth may reduce contact and diminish the client-nurse relationship
5	Abolfotouh et al., [19]	2019	Access smartphone use and perceptions among healthcare workers	Mixed-method (cross-sectional)	*N* = 351 (HCWs)	There is a common use of smartphones among HCWs.
						There are negative perceptions among HCWs on the use of telehealth–practicability
6	Alkamel et al., [22]	2020	To explore stakeholder opinions and HCWs expectations on the use of mobile app	Qualitative-interviews	*N* = 22 (HCWs)	Perceived improvement and efficiency in management
						Better communication
7	Thapa et al., [24]	2021	To identify the willingness to use digital health tools on patients	Quantitative cross-sectional study		87% of nurses and 66% of nursing students, we are willing to use telehealth
8	Al-Marashi & Al-Zghool [25]	2018	To explore the factors influencing job performance among nurses	Mixed-method	*N* = 344 nurses	Lack of managerial effort, relationship with other nurses, fairness of shift work

## Additional Points

*Future Directions*. Saudi Arabia should come up with strategies for promoting health education to encourage its citizens to live healthy lifestyles with the aim of trying to combat the rising prevalence of lifestyle diseases. Again, the Saudi Arabia government should encourage more of its citizens to pursue careers in nursing as well as other health courses to bridge the gap of health professionals which currently faces the country. This will help reduce the high costs involved in importing a health workforce. The findings of this study may aid key professionals in addressing present issues and barriers, as well as prioritizing the most important areas for change.To gain a clearer picture of existing practice and plans for the future of telehealth in Saudi Arabia, more study on the experiences and breadth of telehealth acceptance and adoption levels in diverse geographical areas across the country is recommended in this review.
